# PU.1/microRNA-142-3p targets ATG5/ATG16L1 to inactivate autophagy and sensitize hepatocellular carcinoma cells to sorafenib

**DOI:** 10.1038/s41419-018-0344-0

**Published:** 2018-02-22

**Authors:** Kai Zhang, Jing Chen, Hao Zhou, Ying Chen, Yingru Zhi, Bei Zhang, Longbang Chen, Xiaoyuan Chu, Rui Wang, Chunni Zhang

**Affiliations:** 1 Department of Medical Oncology, Jinling Hospital, School of Medicine, Nanjing University, Nanjing, Jiangsu Province People’s Republic of China; 2 Department of Medical Oncology, Jinling Hospital, School of Medicine, Nanjing Medical University, Nanjing, Jiangsu Province People’s Republic of China; 3 Department of Clinical Laboratory, Jinling Hospital, School of Medicine, Nanjing University, Nanjing, Jiangsu Province People’s Republic of China

## Abstract

Sorafenib is currently the only systemic agent approved for treatment of advanced hepatocellular carcinoma (HCC). However, intrinsic and acquired resistance to sorafenib remains a great challenge with respect to improving the prognoses of patients with HCC. The cyto-protective functions of autophagy have been suggested as a potential mechanism by which chemoresistance or targeted drug resistance occurs in tumour cells. In the present study, miR-142-3p was identified as a novel autophagy-regulating microRNA (miRNA) that plays a vital role in sorafenib resistance in HCC cells. Gain- and loss-of-function assays revealed that ectopic miR-142-3p upregulation sensitized HCC cells to sorafenib by reducing sorafenib-induced autophagy, enhancing sorafenib-induced apoptosis and inhibiting cell growth, whereas miR-142-3p inhibition exerted contrasting effects. Bioinformatics analysis and luciferase reporter and rescue assays showed that autophagy-related 5 (ATG5) and autophagy-related 16-like 1 (ATG16L1) are potential targets through which miR-142-3p regulates autophagy inhibition. Furthermore, we verified that PU.1 regulated the expression of miR-142-3p in conjunction with our cellular experiments and the related results in the literature. Our findings show that targeting the PU.1–miR-142-3p–ATG5/ATG16L1 axis may be a useful therapeutic strategy for preventing cyto-protective autophagy to overcome sorafenib resistance.

## Introduction

Hepatocellular carcinoma (HCC) is one of the leading causes of cancer-related death worldwide. More than 500,000 new patients are diagnosed each year, and half of these patients live in China^1, 2^. The incidence of HCC has almost doubled in developed areas during the past 20 years, and the disease has also become more common in developing areas^3^. Most patients with HCC are diagnosed at advanced disease stages and do not have an opportunity to undergo surgical resection. The efficacies of the treatments for patients with advanced HCC, including systemic chemotherapy, transarterial chemoembolization and ablation, have improved, but the prognosis of the disease remains unsatisfactory. Sorafenib, a multitargeted kinase inhibitor, has greatly revolutionized the treatment of HCC^4, 5^. Unfortunately, the long-term value of sorafenib is limited due to primary and acquired resistance^6^. Thus studies exploring the molecular mechanisms underlying sorafenib resistance, as well as studies aiming to develop new strategies for the treatment of patients with HCC, are urgently needed. Many factors contribute to resistance in HCC; one such factor is autophagy activation^7, 8^.

The findings of many recent studies support the theory that the cyto-protective effects of autophagy in tumour cells represent a mechanism by which treatment resistance occurs^9^. In simple terms, autophagy is a self-cannibalization process that occurs in both normal cells and cancer cells^10^. Autophagosomes, which comprise double-membrane vesicles, generally capture intracellular cytoplasm, damaged organelles, protein aggregates and other pathogens and subsequently fuse with lysosomes, after which the inner contents of the autophagosomes are broken down by proteases, lipases, nucleases and glycosidases. In healthy cells, autophagy contributes to the maintenance of intracellular homeostasis by serving as a garbage removal unit, providing energy during periods of starvation or protecting the cell from exotic invasive agents^11^. Autophagy is a complex process; it plays a dual role in cancer development and cancer treatment. In cancer cells, autophagy has been linked to therapy resistance. However, the mechanism underlying the relationship between autophagy and sorafenib resistance in HCC remains unclear and requires further elucidation. A variety of autophagy-related proteins participate in different stages of autophagy^12^. Recent studies indicate that the autophagy-related 16-like-1 (ATG16L1)–autophagy-related 5 (ATG5)-autophagy-related 12 (ATG12) system plays critical roles in autophagosome formation and elongation. For example, the system functions as novel E3-like enzyme to regulate the site at which microtubule-associated protein light chain 3 (LC3) binds with phosphatidylethanolamine (PE). ATG12 binds to autophagy-related 7 (ATG7) and autophagy-related 10 (ATG10). This complex binds to ATG5, and the resultant ATG12–ATG5 complex binds to ATG16L1^13^. The LC3 family, which includes LC3-I and LC3-II, also participates in this process, as stated above. During this ubiquitin-like response, pro-LC3 is cleaved into LC3-I by ATG4 and then binds to PE along with ATG7 (E1-like enzyme) and ATG3 (E2-like enzyme), resulting in the generation of LC3-II, which is closely associated with autophagosome membrane formation^14^. Thus LC3-II acts as a strong biomarker for autophagy^15^. It is generally accepted that tumour cells utilize autophagy to overcome the stresses caused by chemotherapeutic agents, radiation and molecular-targeted agents, including sorafenib. However, how tumour cells utilize autophagy to induce sorafenib resistance remains unknown and needs to be elucidated further.

MicroRNAs (miRNAs) are small non-coding RNAs that regulate the expression of many genes involved in cell proliferation, differentiation and apoptosis at the posttranscriptional level by binding to the 3’-untranslated region (3’-UTR) of target mRNAs^16–18^. Emerging evidence suggests that miRNAs not only play dual roles in cancer therapy, as they sometimes act as oncogenes, but also sometimes act as tumour suppressors. Thus research regarding the mechanism by which miRNAs regulate autophagy in cancer therapy and resistance has attracted great attention. Reports have identified several autophagy-related miRNAs (e.g., miR-30a, miR-101, miR-181a and miR-375) that decrease autophagic activity to increase the sensitivity of tumour cells to chemotherapeutic or molecular-targeted agents. MiR-142-3p, which is located in human chromosome17q22, has been reported to serve as both an oncogene and a tumour suppressor in multiple human cancers^19–22^. However, the regulatory role of miR-142-3p in sorafenib resistance in HCC cells and the possible mechanism underlying this role are unclear. Here we described the roles of miR-142-3p in the control of autophagy and showed that this miRNA sensitized HCC cells to sorafenib by blocking sorafenib-induced autophagy. We also provided evidence showing that two key autophagy proteins, ATG5 and ATG16L1, are two rate limiting and direct autophagy-related targets of miR-142-3p. A previous study found that miR-142-3p is regulated by a key transcription factor—PU.1. In our study, we also demonstrated that PU.1 modulates miR-142-3p expression. Therefore, PU.1–miR-142-3p–ATG5/ATG16L1 axis dysregulation is heavily involved in the development of sorafenib resistance in HCC.

## Results

### Autophagy is activated in sorafenib-treated HCC cells

**Fig. 1:**
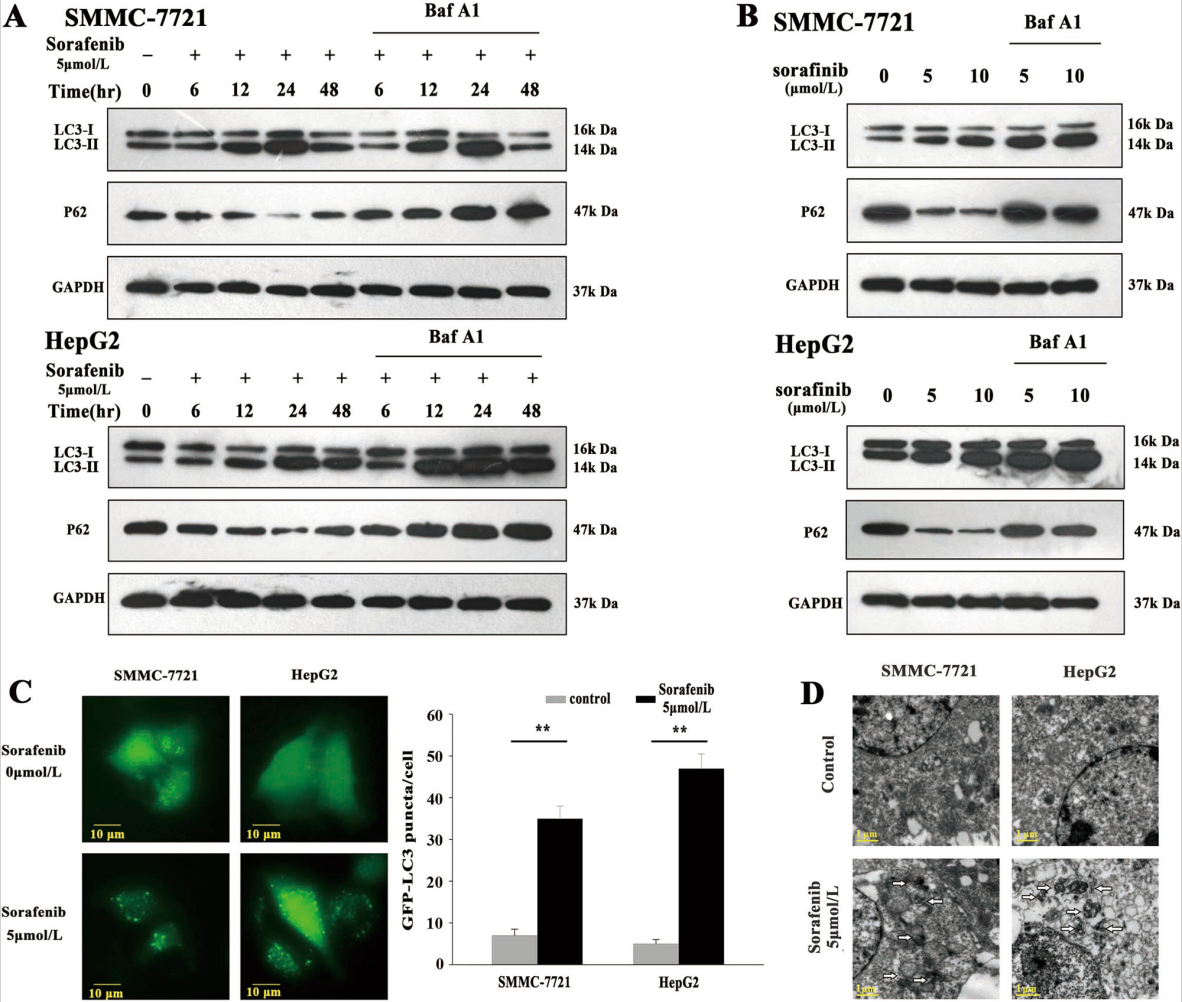
Elevated autophagy occurs in HCC cells treated with sorafenib. **a** Western blotting was performed to measure autophagic activity in HCC cells treated with sorafenib and with or without Baf-A1. **b** Western blotting was performed to measure the autophagy levels in HCC cells treated with increasing concentrations of sorafenib and with or without Baf-A1. **c** SMMC-7721 and HepG2 cells were transfected with a GFP-LC3 plasmid, after which they were treated with the indicated concentrations of sorafenib for 24 h. At the end of treatment, the cells were inspected under a fluorescence microscope. **d** Transmission electron microscopy was utilized to determine the autophagosome levels. All data are presented as the mean ± S.D. from three independent experiments. The *p*-values represent comparisons between groups (**p < 0.01)

Autophagy reportedly occurs under basal conditions but can also be stimulated under various stress conditions, including starvation, hypoxia and treatments. LC3 has emerged as a robust marker of autophagosomes. During autophagy induction, the nonlipidated form of LC3 (LC3-I) is conjugated with PE and then converted into the lipidated form of LC3 (LC3-II). In addition to LC3-II, p62 also serves as a marker of autophagy induction. p62, an adapter protein, is selectively degraded via autophagy. Thus detecting the levels of LC3 and p62 is a common method of monitoring autophagy. The LC3-II levels were further elevated in the presence of bafilomycin A1, a late-phase autophagy inhibitor that prevents autophagosome–lysosome fusion and LC3-II degradation, indicating an increase of autophagic flux in the sorafenib-treated and control SMMC-7721 cells. (Fig. 1a, b). Within the 24 h initation of sorafenib treatment, autophagy levels in the sorafenib-treated HCC cells were clearly increased in a dose- and time-dependent manner compared with those in the control cells; furthermore, the LC3-II levels were higher, and the p62 levels were lower in the sorafenib-treated cells than in the control cells. At 48 h after treatment initiation, the time-dependent differences were eliminated. Additionally, we found that both LC3-II and p62 levels increased in sorafenib-treated HCC cells exposed to the lysosome inhibitor bafilomycin A1 (Baf-A1). Consistent with the above findings pertaining to LC3-II accumulation, the percentage of LC3 puncta-positive cells in the green fluorescent protein (GFP)-positive cell population also increased significantly in sorafenib-treated HCC cells compared with control cells (Fig. 1c). Additionally, we observed that characteristic autophagosomes appeared in sorafenib-treated HCC cells (Fig. 1d). Taken together, these data demonstrate that sorafenib treatment increases autophagy activity in HCC cells.

### Autophagy inhibition enhances the sensitivity of HCC cells to sorafenib

**Fig. 2:**
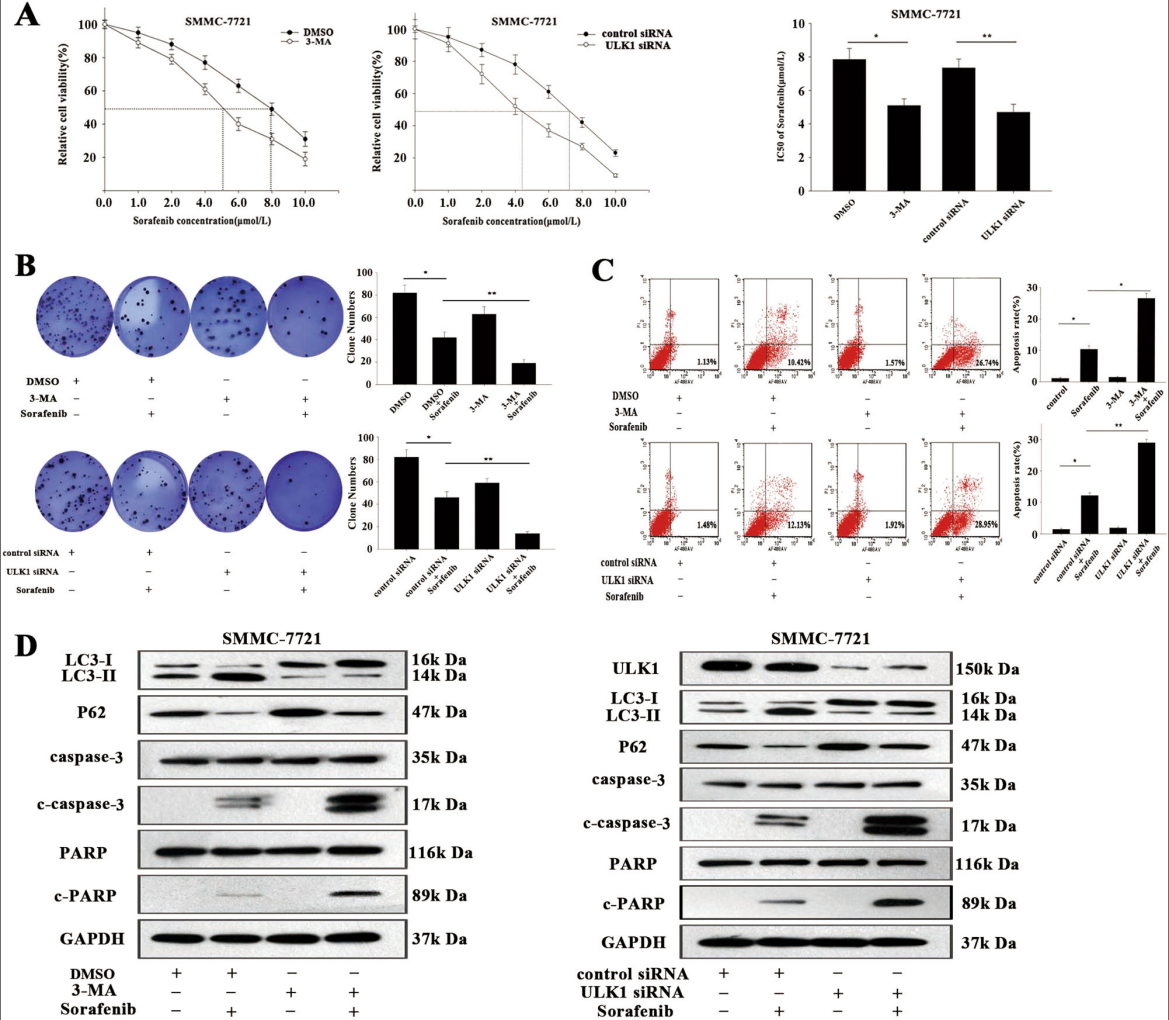
Suppression of autophagy enhanced the sensitivity of HCC cells to sorafenib. **a** MTT assay was employed to measure the relative viability of SMMC-7721 cells treated with sorafenib after treatment with 3-MA or ATG1/ULK1 silencing. **b**–**e** Flow cytometric and western blotting were performed to measure the apoptosis rate and apoptosis (c-caspase3 and c-PARP)- and autophagy-related protein levels. All data are presented as the mean ± S.D. from three independent experiments. The *p*-values represent comparisons between groups (*p < 0.05, **p < 0.01)

To confirm that autophagy protects against sorafenib toxicity in HCC cells, we investigated whether autophagy inhibition enhances the sensitivity of HCC cells to sorafenib using 3-methyladenine (3-MA, an autophagy inhibitor) or small interfering RNA (siRNA) targeting ULK1, which is a critical molecule in autophagy formation. MTT (3-[4,5-dimethylthiazol-2-yl]-2,5 diphenyl tetrazolium bromide) assay results revealed that the responses of HepG2 and SMMC-7721 cells to sorafenib were significantly restored after 3-MA or ULK1/siRNA treatment (Fig. 2a; Fig. S1A). Additionally, treatment with 3-MA or ULK1/siRNA with or without sorafenib markedly blunted autophagy activity in HCC cells, leading to decreased colony-formation capacity, increased apoptosis and higher protein expression levels of cleaved (c)-caspase 3 and c-poly ADP-ribose polymerase (c-PARP) (Fig. 2b–d; Fig. S1B-D). Collectively, these data showed that autophagy inhibition increased the sensitivity of HCC cells to sorafenib.

### miR-142-3p inhibits autophagy during sorafenib treatment and is upregulated by PU.1 in HCC cells

**Fig. 3:**
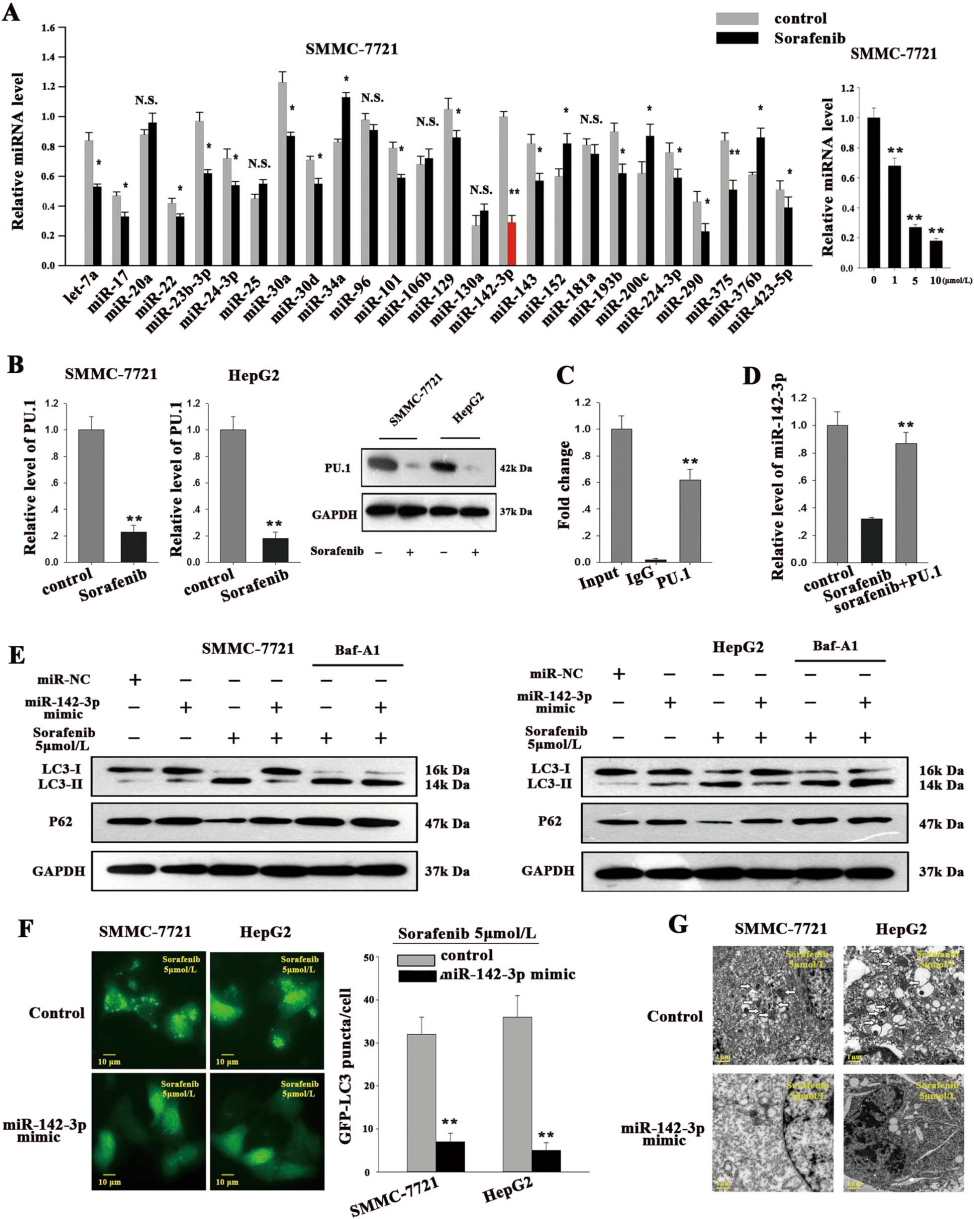
MiR-142-3p may inhibit autophagy during sorafenib treatment and may be upregulated by PU.1 in HCC cells. **a** qRT-PCR was used to assess autophagy- and apoptosis-related miRNA levels in SMMC-7721 cells treated with sorafenib. **b** qRT-PCR and western blotting were employed to determine PU.1 levels in HepG2 and SMMC-7721 cells treated with sorafenib. **c** ChIP assay was used to confirm that PU.1 interacts with miR-142-3p. **d** Transfection with PU.1 expression vectors reversed the suppressive effects of sorafenib on miR-142-3p. **e** LC3 and p62 levels were measured in cells treated with miR-142-3p mimics (left) or (inhibitor) after sorafenib treatment. **f**, **g** The levels of sorafenib-induced LC3 puncta (**f**)  and autophagic vacuoles (**g**) were counted after treatment with miR-142-3p mimics or inhibitors. All data are presented as the mean ± S.D. from three independent experiments. The *p*-values represent comparisons between groups (*p < 0.05, **p < 0.01)

It was recently reported that miRNAs play roles in regulating autophagy by modulating the expression of key autophagy-related proteins. We performed TaqMan PROBE-BASED real-time quantitative reverse-transcription polymerase chain reaction (qRT-PCR) assays to detect the expression of a panel of miRNAs reported to be associated with cellular autophagy or apoptosis. In particular, we were interested in studying miR-142-3p. As shown in Fig. 3a, among the 22 miRNAs tested, miR-142-3p displayed the largest decrease in its expression in SMMC-7721 cells following sorafenib treatment. A similar reduction in miR-142-3p expression was observed in HepG2 cells after sorafenib treatment (Fig. S2A). The transcription factor PU.1 was previously identified as a critical regulator of miR-142-3p expression, and one study identified one binding motif for PU.1 within the region from −199 to −193 in the miR-142 promoter^23^. Thus we investigated whether sorafenib treatment downregulated miR-142-3p expression by regulating PU.1 expression. As shown in Fig. 3b, sorafenib treatment significantly reduced PU.1 mRNA and protein expression levels in HCC cells. We then subcloned the miR-142-3p promoter into a pGL3-basic vector (pGL3/miR-142-pro) and co-transfected the miR-142 promoter luciferase reporter construct and pcDNA/PU.1 into SMMC-7721 and HEK293T cells. We observed that co-transfection with pcDNA/PU.1 triggered the luciferase activity driven by the miR-142 promoter (Fig. S2B). A chromatin immunoprecipitation (ChIP) assay was performed to determine whether PU.1 was recruited to the binding site. We observed that in immunoprecipitates from SMMC-7721 and HepG2 cells, the 248-bp DNA fragment was amplified by PU.1 antibody but not by control immunoglobulin G (IgG) antibody (Fig. 3c; Fig. S2C), suggesting that endogenous PU.1 binds to the region −199 to −193 bp upstream of the miR-142 promoter. In addition, transfection of the pcDNA/PU.1 vector reversed the decreases in miR-142 expression in both sorafenib-treated cell lines (Fig. 3d; Fig. S2D). These results indicated that PU.1 is responsible for inhibiting sorafenib-induced miR-142-3p inhibition in HCC cells. To investigate the functional role of miR-142-3p in sorafenib-induced autophagy, we established SMMC-7721 and HepG2 cells transiently expressing miR-142-3p or miR-NC by transfecting the cells with miR-142-3p or miR-NC mimics (Fig. S2E). Exposing SMMC-7721/miR-NC or HepG2/miR-NC cells to sorafenib increased autophagic flux, a change reflected by enhancements of LC3-II expression. However, miR-142-3p overexpression partly reversed sorafenib-induced LC3-II accumulation in HCC cells (Fig. 3e). In the absence of Baf-A1, the LC3-II levels could be reduced by miR-142-3p, but this decrease could be reversed by the introduction of Baf-A1 in the HCC cells treated with sorafenib. These results demonstrate that reductions in LC3-II expression do not result from enhanced LC3-I degradation. In contrast, treatment with antagomir-142-3p enhanced sorafenib-mediated autophagic flux, as shown in Fig. S3E. Moreover, we found that miR-142-3p/mimic suppressed the formation of sorafenib-mediated LC3 puncta (Fig. 3f) and autophagic vacuoles (Fig. 3g). These data suggest that miR-142-3p functions as a negative regulator of sorafenib-mediated autophagy in HCC cells.

### Restored miR-142-3p expression re-sensitizes HCC cells to sorafenib by inhibiting autophagy

**Fig. 4:**
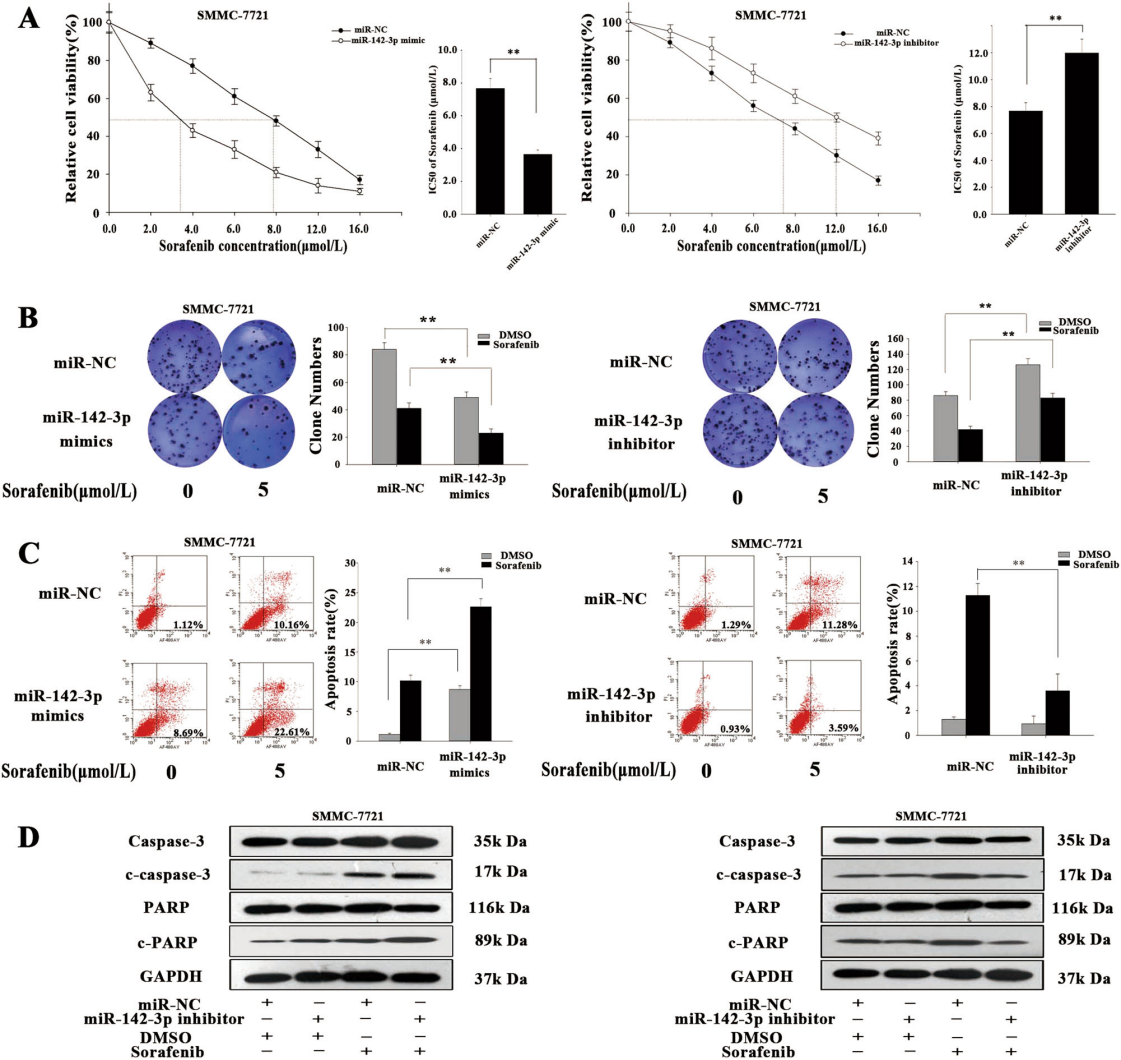
Forced miR-142-3p expression re-sensitized HCC cells to sorafenib via autophagy inhibition. **a**,** b** MTT and colony-formation assays were performed to measure the influence of miR-142-3p on the cytotoxicity of different concentrations of sorafenib and SMMC-7721 cell proliferation. **c** Flow cytometric analysis was employed to determine the effect of miR-142-3p on SMMC-7721 apoptosis rates in cells treated with different concentrations of sorafenib. **d** Western blotting was applied to assess apoptosis- and autophagy-related protein levels. All data are presented as the mean ± S.D. from three independent experiments. The *p*-values represent comparisons between groups **p < 0.01)

We next sought to investigate the mechanism by which miR-142-3p impacts the sensitivity of HCC cells to sorafenib. MTT and colony-formation assays showed that transfection of miR-142-3p/mimic significantly improved cytotoxicity and inhibited proliferation in a dose-dependent manner in sorafenib-treated SMMC-7721 cells, whereas transfection of miR-142-3p/inhibitor had contrasting effects (Fig. 4a, b). Additionally, flow cytometric analysis indicated that miR-142-3p re-expression clearly increased apoptosis in a dose-dependent manner in sorafenib-treated SMMC-7721 cells, whereas miR-142-3p inhibition had contrasting effects (Fig. 4c). In addition, we performed western blotting assay to detect apoptosis-related protein expression (Fig. 4d). miR-142-3p re-expression increased the protein expression levels of c-caspase-3 and c-PARP in sorafenib-treated SMMC-7721 cells compared with control cells, while inhibition of miR-142-3p decreased the expression levels of those proteins in sorafenib-treated cells compared with control cells. Similar results were observed in the experiments involving HepG2 cells (Fig. S4A-D). Therefore, our findings show that miR-142-3p overexpression increases the sensitivity of cancer cells to sorafenib treatment by inhibiting autophagy.

### ATG5 and ATG16L1 are direct targets of miR-142-3p

**Fig. 5:**
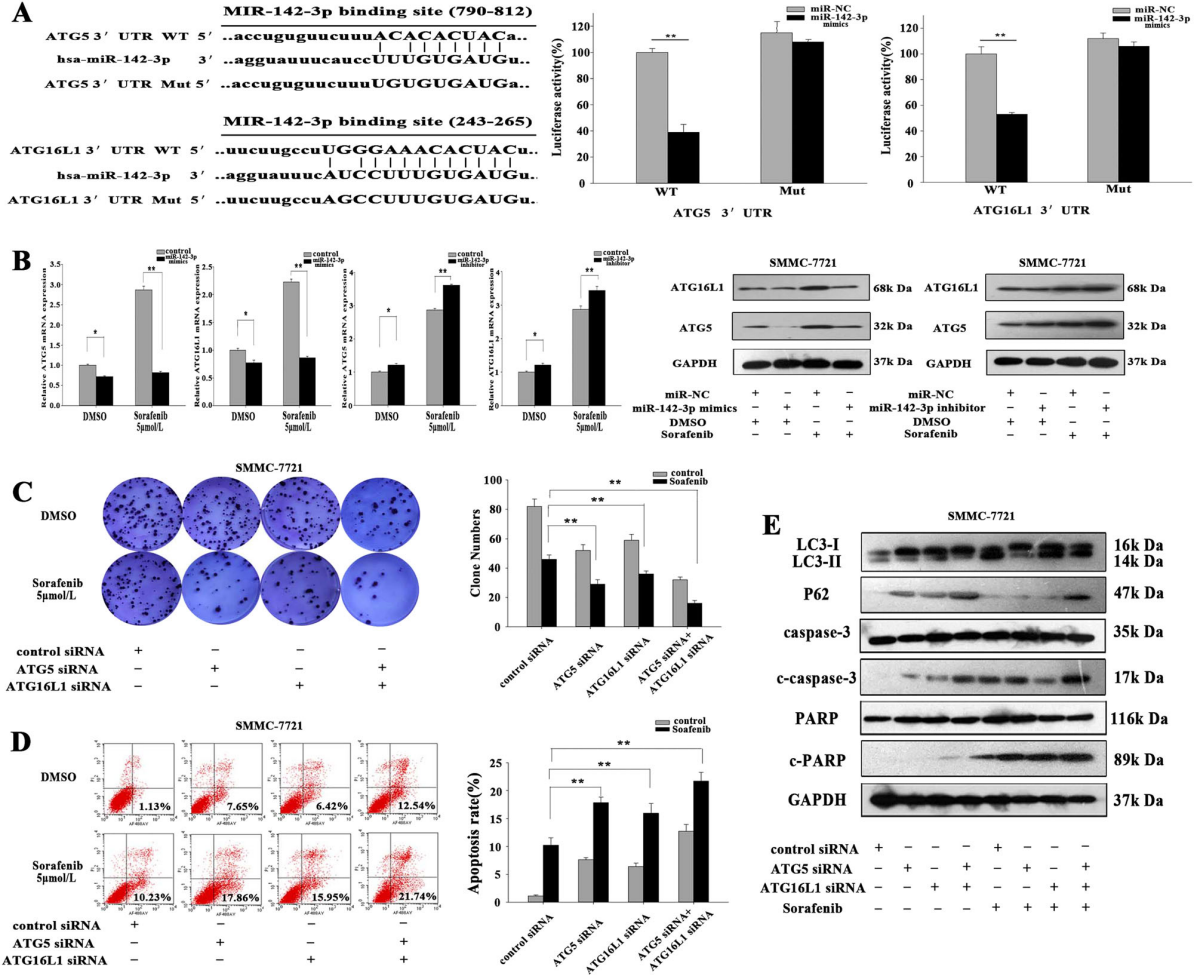
ATG5 and ATG16L1 are the direct targets of miR-142-3p. **a** The binding sites for miR-142-3p in the 3’-UTRs of ATG5 and ATG6 (left) were determined, and luciferase reporter assays were conducted to confirm the relationships between miR-142-3p and ATG5 and ATG16L1. **b** qRT-PCR and western blotting were used to measure ATG5 and ATG16L1 mRNA and protein levels in SMMC-7721 cells transfected with miR-142-3p mimics or inhibitors and treated with or without sorafenib. **c**, **d** Colony-formation assay and flow cytometric analysis were employed to measure the effects of ATG5 and ATG16L on proliferation and apoptosis in SMMC-7721 cells treated with or without sorafenib. **e** Western blotting was performed to measure apoptosis- and autophagy-related protein levels. All data are presented as the mean ± S.D. from three independent experiments. The *p*-values represent comparisons between groups (*p < 0.05, **p < 0.01)

To elucidate the mechanism by which miR-142-3p inhibits autophagy, we searched for autophagy-related genes containing potential binding sites for miR-142-3p in their 3’-UTRs using two publicly available bioinformatics tools (miRanda and TargetScan). ATG5 (GenBank accession number: NM_004849.2, 790-812) and ATG16L1 (GenBank accession number: NM_001190267.1, 243-265) were predicted as targets of miR-142-3p and the latter was verified by Kasandra J. Riley’s study^24^. The predicted interactions between miR-142-3p and the 3’-UTRs of ATG5 and ATG16L1 are shown in Fig. 5a. To confirm that these genes are targets, we constructed 3’-UTR reporters of ATG5 and ATG16L1 containing putative miR-142-3p binding sites or mutant binding sites downstream of the luciferase reporters. We then co-transfected human HEK 293T cells with the reporter constructs and the miR-142-3p/mimic or miR-NC/mimic. We performed luciferase activity assay 48 h after transfection. As shown in Fig. 5a, co-transfection of HEK 293T cells with the miR-142-3p/mimic and the wild-type reporter construct significantly decreased luciferase activity levels, whereas co-transfection of HEK 293T cells with a reporter containing point mutations at the putative miR-142-3p-binding sites did not affect luciferase activity, indicating that miR-142-3p interacts directly with ATG5 or ATG16L1 mRNA. As shown in Fig. 5b, transfection of miR-142-3p/mimic significantly decreased ATG5 and ATG16L1 mRNA and protein expression levels in SMMC-7721 cells treated with or without sorafenib, while transfection of miR-142-3p/inhibitor increased ATG5 and ATG16L1 mRNA and protein expression levels in cells treated with or without sorafenib. Colony formation and flow cytometry assays indicated that siRNA-mediated downregulation of ATG5 or ATG16L significantly inhibited proliferation and enhanced apoptosis in SMMC-7721 cells treated with sorafenib (Fig. 5c, d). Western blotting revealed that the expression levels of the indicated apoptosis-related proteins (c-caspase-3 and c-PARP) were increased in ATG5 siRNA- and ATG16L1 siRNA-transfected SMMC-7721 cells, particularly in sorafenib-treated cells, compared with control cells. The expression levels of the autophagy-related protein LC3II were higher while those of p62 were lower in ATG5 siRNA and ATG16L1 siRNA cells than the respective levels in control cells. These changes were partially attenuated in cells transfected with ATG5 siRNA or ATG16L1 siRNA (Fig. 5e). Similar results were observed in the experiments involving HepG2 cells (Fig. S3E-H). These results demonstrate that miR-142-3p downregulates ATG5 and ATG16L1 mRNA by directly interacting with their 3’-UTRs.

### The effect of miR-142-3p on the sensitivity of HCC cells to sorafenib is dependent on ATG5 and ATG16L1 regulation

**Fig. 6:**
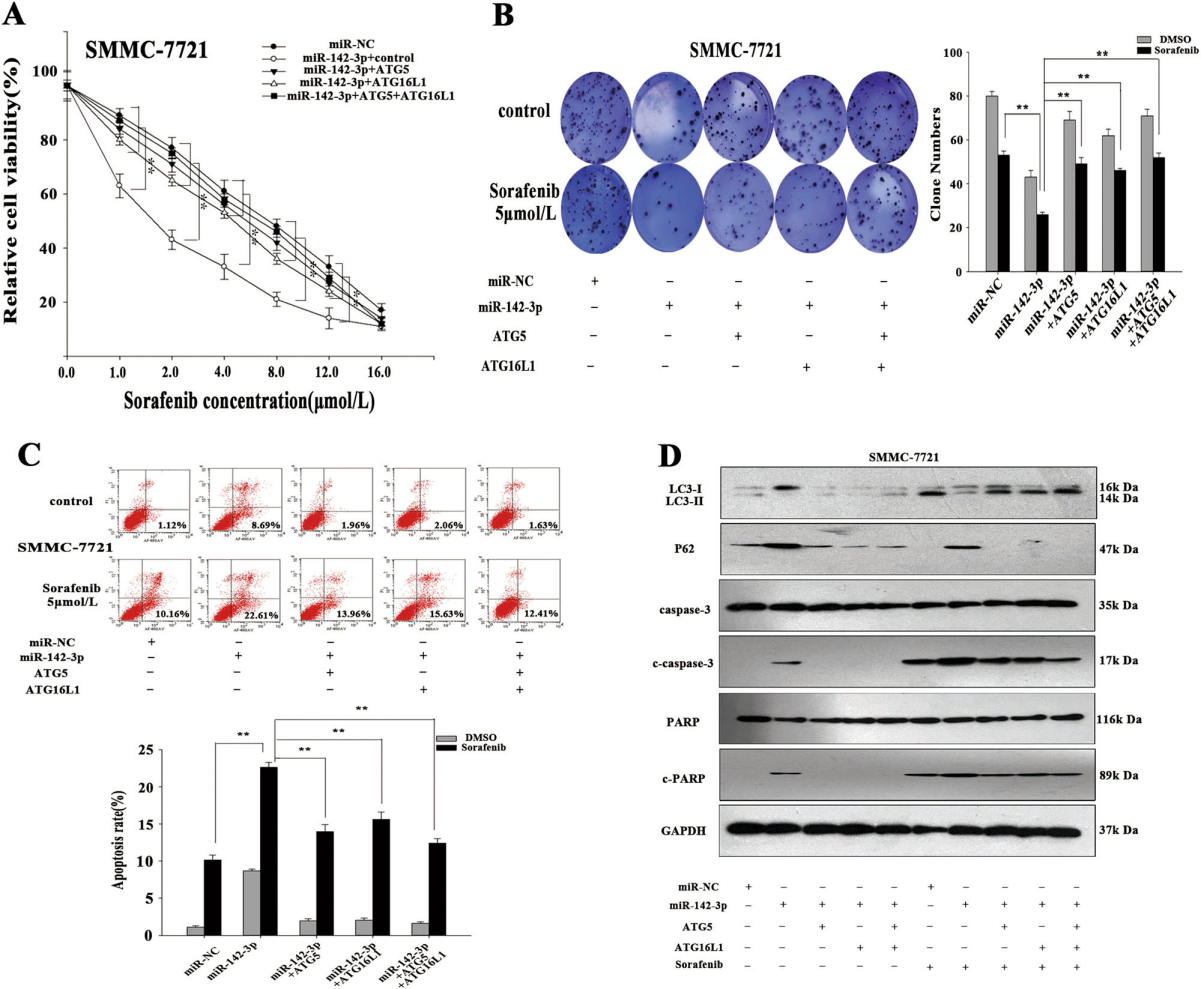
The function of miR-142-3p in sorafenib resistance is dependent on the regulation of ATG5 and ATG16L1. **a** MTT assay was performed to measure the sensitivity of SMMC-7721 cells co-transfected with miR-142-3p mimics and ATG5 or ATG16L1 to sorafenib. **b** Colony-formation assay was employed to determine the proliferation ability of SMMC-7721 cells co-transfected with miR-142-3p mimics and ATG5 or ATG16L1. **c** Flow cytometric assay was employed to measure the apoptosis rate in SMMC-7721 cells co-transfected with miR-142-3p mimics and ATG5 or ATG16L1. **d** Western blotting was utilized to assess the levels of the apoptosis-related proteins c-caspase-3 and c-PARP and autophagy-related proteins in SMMC-7721 cells co-transfected with miR-142-3p mimics and ATG5 or ATG16L1. All data are presented as the mean ± S.D. from three independent experiments. The *p*-values represent comparisons between groups (**p < 0.01)

To confirm that ATG5 or ATG16L1 downregulation was responsible for the effects of miR-142-3p on HCC cell sensitivity to sorafenib, we performed “rescue assays” in which ATG5 or ATG16L1 was overexpressed in a plasmid lacking the miR-142-3p response element. miR-142-3p overexpression significantly reduced the levels of endogenous ATG5 and ATG16L1; however, cells co-transfected with the miRNA and a plasmid containing either ATG5 or ATG16L1 expressed ATG5 or ATG16L1, respectively, at near-physiological levels (Fig. S4A and B). The MTT and colony-formation assay results indicated that the re-introduction of either ATG5 or ATG16L1 in the presence of miR-142-3p reversed the inhibition of SMMC-7721 cell growth induced by the combination of the miRNA and sorafenib (Fig. 6a, b). Furthermore, flow cytometry was performed to detect the apoptosis rate in SMMC-7721 cells co-transfected with miR-142-3p mimics and ATG5 or ATG16L1. We observed that the increases in apoptosis mediated by miR-142-3p were partially abolished by ATG5 and ATG16L1 (Fig. 6c). Moreover, western blotting showed that the increases in the expression levels of the indicated apoptosis-related proteins (c-caspase-3 and c-PARP) induced by miR-142-3p were partially attenuated by the re-introduction of ATG5 or ATG16L1 (Fig. 6d). Meanwhile, the decreases in autophagy levels induced by miR-142-3p were partially reversed by ATG5 or ATG16L1 (Fig. 6d). Similar phenomena were observed in the experiments involving HepG2 cells (Fig. S4C-F). These findings revealed that the effects of miR-142-3p on sorafenib resistance in HCC cells are ATG5 and ATG16L1 dependent.

### Restored miR-142-3p expression enhances the in vivo anti-tumour effects of sorafenib in HCC

**Fig. 7:**
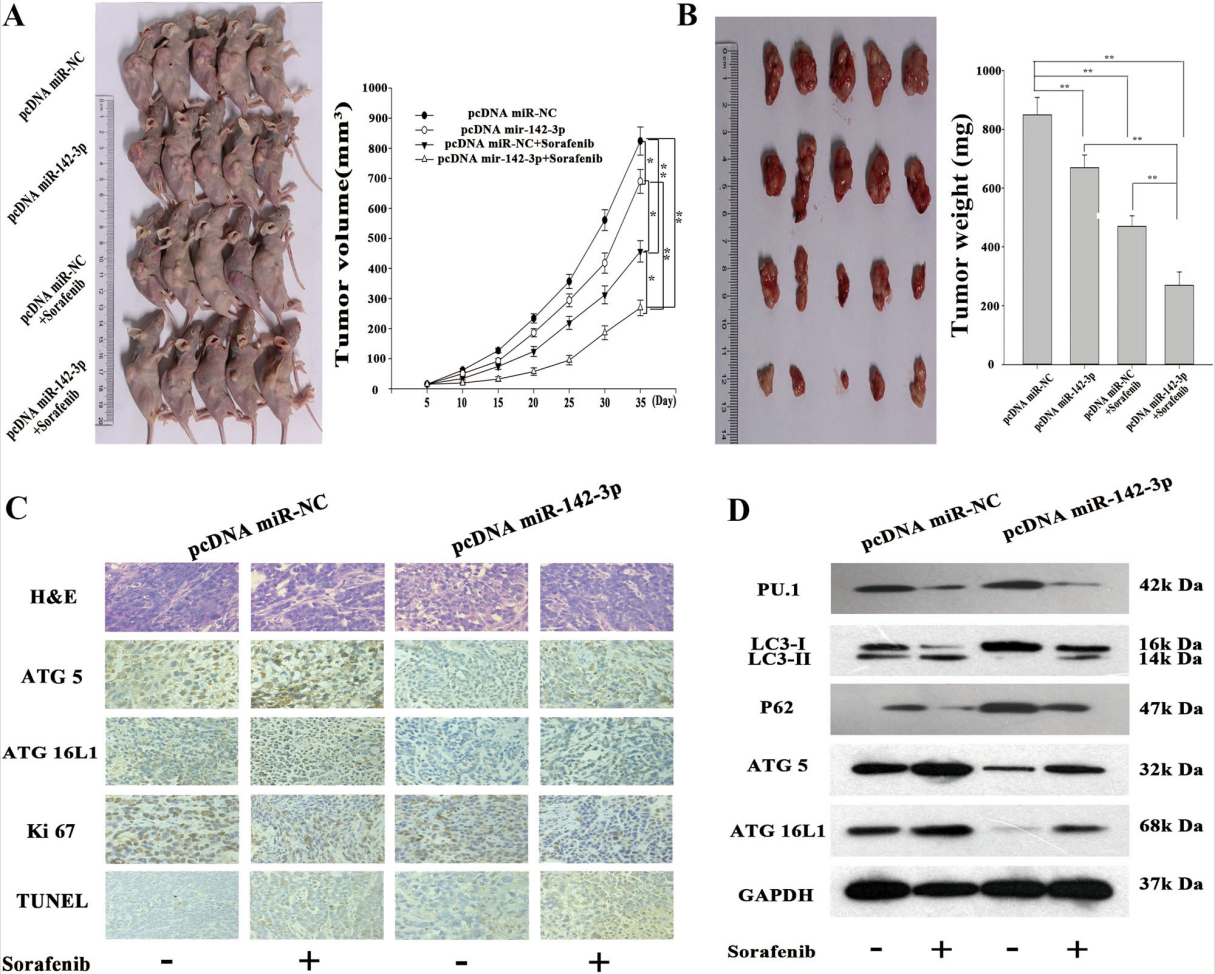
MiR-142-3p enhanced the anti-tumour effects of sorafenib in HCC in vivo. **a** Growth curves for tumour volumes and representative photographs of tumour-bearing mice. **b** Representative photographs of tumour and tumour weight. **c** ATG5 and ATG16L1 expression levels were analysed by immunostaining in HepG2/miR-142-3p and HepG2/control xenografts after sorafenib therapy. Immunostaining and TUNEL staining were employed to measure PCNA and Ki67 levels, as well as apoptosis rates, in tumours derived from HepG2/miR-142-3p and HepG2/control xenografts after sorafenib therapy. **d** Western blotting was performed to assess LC3, ATG5, ATG16L1 and p62 protein expression in tumours derived from HepG2/miR-142-3p and HepG2/control xenografts after sorafenib therapy. All data are represented as the mean ± S.D. from three independent experiments. The *p*-values represent comparisons between groups (*p < 0.05, **p < 0.01)

To investigate the effect of miR-142-3p on the sensitivity of HCC cells to sorafenib in vivo, we stably transfected HepG2 cells with miR-142-3p plasmid constructs (pcDNA/miR-142-3p) or control plasmids (pcDNA/miR-NC). In these experiments, mice with tumours were treated with sorafenib, and tumour volumes were measured every other day for 2 weeks. As shown in Fig. 7a, b, tumours derived from mice inoculated with HepG2/miR-142-3p were more sensitive to sorafenib therapy than tumours derived from mice inoculated with HepG2/control. However, there were no differences in tumour growth between HepG2/control and HepG2/miR-142-3p tumours treated without sorafenib. Additionally, immunostaining analysis showed that ATG5 and ATG16L1 expression levels were significantly decreased in the HepG2/miR-142-3p xenografts compared with the HepG2/control tumour xenografts after sorafenib treatment (Fig. 7c). Immunostaining analysis and terminal deoxinucleotidyl transferase-mediated dUTP-fluorescein nick end labelling (TUNEL) staining revealed that the rates of proliferating cell nuclear antigen (PCNA) and Ki67 positivity were lower, and the rate of apoptosis was higher in tumours derived from HepG2/miR-142-3p cells than in tumours derived from HepG2/control cells following sorafenib treatment (Fig. 7c). We also assessed LC3, ATG5, ATG16L1 and p62 protein expression levels in tumours derived from HepG2/miR-142-3p and HepG2/control xenografts after sorafenib therapy (Fig. 7d). These results highlight that miR-142-3p plays a critical role in regulating the in vivo sensitivity of HCC cells to sorafenib.

### MiR-142-3p expression is positively correlated with PU.1 expression and negatively correlated with ATG5/ATG16L1 expression in HCC tissues

**Fig. 8:**
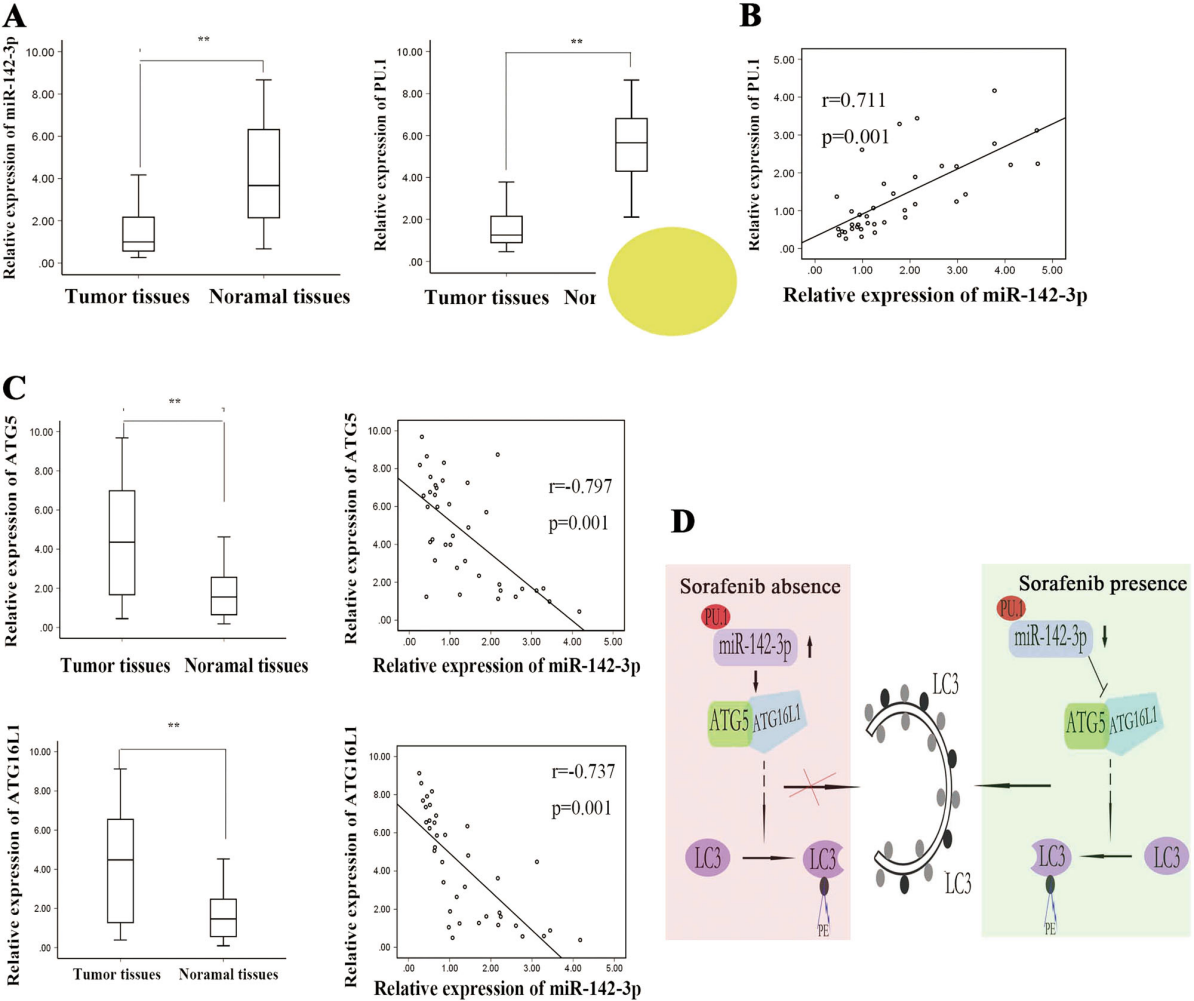
miR-142-3p expression was positively correlated with PU.1 expression and negatively correlated with ATG5/ATG16L1 expression in HCC tissues. **a** miR-142-3p, PU.1, ATG5 and ATG16L1 levels in 38 HCC tissues and matched normal tissues were measured by qRT-PCR. **b** The correlation between miR-142-3p and PU.1 expression was analysed. **c** The correlations between miR-142-3p and ATG5 and ATG16L1 expression were analysed. **d** Schematic overview of miR-142-3p/ATG5/ATG16L1 regulatory signalling. All data are presented as the mean ± S.D. from three independent experiments. The *p*-values represent comparisons between groups (**p < 0.01)

The expression of the above four molecules was analysed in 38 samples obtained from patients with HCC. As shown in Fig. 8a, miR-142-3p and PU.1 expression levels were significantly decreased in HCC tissues compared with normal tissues, whereas ATG5 and ATG16L1 levels were clearly increased in HCC tissues compared with normal tissues. The results of the correlation analysis showed that PU.1 levels were positively correlated with miR-142-3p levels (two-tailed Spearman’s correlation, *r* = -0.711, *p = *0.000, Fig. 8b) and that ATG5 and ATG16L1 expression levels were negatively correlated with miR-142-3p expression levels (two-tailed Spearman’s correlation, *r* = −0.797, *p = *0.000; and *r* = −0.737, *p = *0.000, Fig. 8c). Collectively, these data indicated that the PU.1/miR-142-3p/ATG5/ATG16L1 axis is involved in regulating the sensitivity of HCC cells to sorafenib (Fig. 8d).

## Discussion

Sorafenib is the first oral medicine approved for the treatment of advanced liver cancer and inhibits the growth of tumour cells and angiogenesis by suppressing the activation of a variety of silk/threonine kinases and tyrosine kinases (such as B-Raf and vascular endothelial growth factor receptor)^25–27^. However, HCC cells can respond to sorafenib in different ways, as they may be prevented from undergoing cell death due to the activation of survival pathways, such as autophagy. These survival pathways restrict sorafenib-induced cell death and induce the development of sorafenib resistance.

In recent years, a large number of studies have described autophagy abnormalities in many human tumours. Autophagy plays an important role in all stages of tumour development, and the relationship between autophagy and tumourigenesis may be twofold. On the one hand, during the early stages of tumourigenesis, an autophagy disorder can increase the instability of the genome and promote carcinogenesis; during the rapid tumour growth and metastasis phases, autophagy can resist the stress conditions and inhibit anoikis to maintain tumour cell survival. On the other hand, autophagy-deficient tumour cells can easily undergo necrosis; however, inflammatory response caused by a necrosis will promote tumour growth and invasion. It has been demonstrated that sorafenib induces autophagy, leading to cell death or survival, depending largely on the complex integration of crosstalk between different intracellular signals. On one hand, autophagy induces cell resistance and leads to reduced apoptosis. On the other hand, protein interactions or nucleic acid molecules can alter the induction of autophagy in response to different cellular pathways by sorafenib. Therefore, its activation changes may help to increase the sensitivity to sorafenib^28^. Autophagy is a process that maintains conserved HCC cell homeostasis following targeted therapy by promoting cell survival. This process also induces resistance in a series of cancer cells^29–33^. The autophagy inhibitors that enhance drug sensitivity to anticancer therapies are being evaluated in clinical research studies. Hence, changes in their activation may be useful to increase sensitivity to sorafenib. Modulating autophagy by regulating the expression of miRNAs is an important new and effective way of resensitizing cancer cells to therapeutics^34–36^. Reports have shown that several miRNAs regulate autophagy by targeting autophagy-related genes in a variety of human cancers, including leukaemia, breast cancer and small cell lung cancer; however, only limited data describing the relationship between miRNAs and autophagy formation in HCC cells are available. In this study, we were interested in the role of miRNA in the autophagy-mediated regulation of sorafenib resistance in HCC cells. First, we identified that HCC cells treated with sorafenib displayed higher autophagy expression levels than HCC cells treated without sorafenib. Moreover, using RT-PCR, we confirmed that a set of miRNAs were significantly differentially expressed between HCC cells treated with and without sorafenib. According to previous reports, some of these differentially expressed miRNAs are related to tumour behaviour.

Here we assessed miR-142-3p as a new miRNA regulating sorafenib-induced autophagy. Our findings demonstrated that sorafenib significantly reduced miR-142-3p levels and that sorafenib exerted its effects by acting on the transcription factor PU.1, which has been identified as a critical factor in miR-142-3p expression. miR-142-3p overexpression led to a significant decrease in the intracellular levels of the following two key autophagy-related proteins: ATG5 and ATG16L1. We demonstrated that miR-142-3p acted on target sequences located in the 3’-UTR regions of ATG5 and ATG16L1 mRNA. In addition, the inhibition of endogenous miR-142-3p by specific antagomirs caused an increase in the expression of these proteins in cells. Upregulating ATG5 or ATG16L1 antagonized the autophagy-blocking effects of miR-142-3p, indicating that miR-142-3p regulates autophagy in HCC cells by modulating ATG5 and ATG16L1 expression. Interestingly, the expression of endogenous miR-142-3p was increased in response to sorafenib treatment. miR-142-3p overexpression following autophagy may limit the autophagic response and prevent the harmful effects of uncontrollable autophagy, such as autophagic cell death. In conclusion, our study found that a new microRNA, miR-142-3p, is important in mammalian autophagy regulation.

Previous studies have shown that miR-142-3p, ATG5 and ATG16L1 impact autophagy-induced cell cytotoxicity. ATG5, ATG12 and ATG16L have been reported to exist mainly as a complex in the mammalian cytoplasm, suggesting that ATG5–ATG12–ATG16L complexes do not lead to the formation and activation of autophagic vacuoles. During autophagic membrane extension, part of the ATG5–ATG12–ATG16L complex is located in the membrane surface. However, the complex dissociates from the membrane after the generation of the double-membrane structure. Therefore, the ATG5–ATG12–ATG16L complex is essential for the extension of the autophagic membrane. The targets of miR-142-3p, ATG5 and ATG16L1, are indispensable proteins in the autophagy pathway. Previous studies have shown that decreases in either ATG5 or ATG16L1 expression were sufficient to inhibit autophagy in a variety of experimental systems^37–42^. Similarly, miR-142-3p-induced decreases in the cellular levels of these proteins had a significant suppressive effect on autophagy in our study.

Mechanistic studies demonstrated that aberrantly regulated miR-142-3p-induced autophagy promotes cell survival by inhibiting the intrinsic apoptotic pathway. Bax is a pro-apoptotic protein that belongs to the Bcl-2-family and exists in the cytosol but translocates to the mitochondria during the induction of apoptosis. Bax induces the release of cytochrome c and the activation of caspase-9 and -3 both in vivo and in vitro. According to previous reports, the release of cytochrome c was related to the opening of the mitochondrial permeability transition pore, an event associated with the dysregulation of the mitochondrial inner transmembrane potential (DCm). We demonstrated that treatment with the miR-142-3p mimic, as well as decreased ATG5 and ATG16L1 expression, promoted these mitochondria-related apoptotic events. In contrast, antagomir-142-3p upregulated autophagy and attenuated the loss of DCm and the activation of caspase-9 during sorafenib treatment, indicating that autophagy protects against apoptosis^43^. In conclusion, autophagy is a process that enables cells to adjust to environmental stresses and protects sorafenib-treated HCC cells from death. Our data suggest that miR-142-3p downregulation induces ATG5- and ATG16L1-dependent autophagy, which induces sorafenib resistance in HCC cells. The molecular mechanism underlying this phenomenon partly involves the regulation of the mitochondria-dependent intrinsic apoptotic pathway. The above results have provided us with a new understanding of the mechanisms underlying HCC cell survival following sorafenib treatment. Further research is necessary to determine the side effects of interfering with miR-142-3p regulation in animals or humans, identify other miR-142-3p target genes, determine the roles of these proteins in the regulation of sorafenib resistance and elucidate the connections between autophagy and other drug-resistance mechanisms.

## Materials and methods

### Patients

Total 38 human HCC tissues and adjacent normal tissues were obtained from the Department of Hepatobiliary Surgery of First Hospital Affiliated to the Chinese PLA General Hospital and the Liver Disease Center of the 81th Hospital of PLA during 2010–2013. Informed consent forms were obtained from all patients prior to surgery. Samples were immediately snap-frozen and stored in liquid nitrogen until use.

### Cell lines

The cell lines HepG2 and SMMC-7721 were cultured in Dulbecco’s modified Eagle’s medium (GIBCO-BRL) supplemented with 10% foetal bovine serum, streptomycin (100 μg/ml) and penicillin (100 U/ml). All cells were fostered at 37 °C in an atmosphere containing 5% CO_2_. All the cell lines were obtained from the Cell Bank of the Chinese Academy of Sciences (Shanghai, China) where they were characterized by mycoplasma detection, DNA–Fingerprinting, isozyme detection and cell vitality detection.

### Real-time quantitative reverse-transcription polymerase chain reaction

Total RNA was extracted from tissues using TRIzol® reagent (Takara, Dalian, China) according to the manufacturer’s instruction. Reverse transcription was performed with PrimeScript RT reagent Kit (Takara,Japan) according to the manufacturer’s protocol. qRT-PCR was performed with SYBR Prime Script RT-PCR Kits (Takara, Japan) based on the manufacturer’s instructions. The PU.1 or miRNAs level was calculated with the 2^−^^ΔΔCt^ method, which was normalized to glyceraldehyde 3-phosphate dehydrogenase (GAPDH) mRNA or U6 rRNA, respectively. All assays were performed in triplicate. The expression levels were relative to the foldchange of the corresponding controls, which were defined as 1.0.

### Cell transfection

Hsa-miRNA-142-3p mimic/negative control mimic and hsa-miRNA-142-3p inhibitor/negative control inhibitor were purchased from Applied Biological Materials (ABM, Canada). Transfections were performed using the Lipofectamine 2000 Kit (Invitrogen) according to the manufacturer’s instructions.

### Dual luciferase reporter assay

ATG5 or ATG16L1 3’-UTR luciferase reporter gene plasmid was constructed, and the fragment containing putative binding sites for miR-142-3p was amplified. Plasmids named pATG5-WT (and pATG5-Mut) or pATG16L1-WT (and pATG16L1-Mut) were generated via subcloning downstream of the luciferase vector. Luciferase reporter experiments were performed in 96-well plates using a DualLuciferase Reporter Assay Kit (Dual-Glo Luciferase Assay System; Promega, Madison, USA) and the SpectraMax M5 instrument software (Molecular Devices, Sunnyvale, USA) to analyse the results. Cells were co-transfected with 100 ng of pATG5-miR-142-3p-UTR-WT or pATG5-miR-142-3p-UTR-Mut in the presence of 50 nM Lipofectamine2000 (Life Technologies, Carlsbad, USA). Likely, cells were co-transfected with 100 ng of pATG16L1-miR-142-3p-UTR-WT or pATG16L1-miR-142-3p-UTR-Mut in the presence of 50 nM Lipofectamine 2000 (Life Technologies, Carlsbad, USA). After 48 h, cells were assayed using a Dual-Luciferase Reporter Assay Kit (Promega) according to the manufacturer’s instructions.

### Colony-formation assay

Clonogenic cells were seeded at a density of 500 cells per well in six-well plates and incubated for 12 days. Cells were subsequently stained with 0.5% crystal violet. The clone formation rate was determined. Independent experiments were conducted in triplicate.

### ChIP assay

ChIP assay was performed with Immunoprecipitation Assay Kits (Millipore) according to the manufacturer’s instructions. Briefly, cells were crosslinked with 1% formaldehyde for 10 min at 37 °C. The cells were then resuspended in 200 μl of lysis buffer and incubated for 10 min on ice. The lysate was sheared to lengths between 200 and 1000 bp by sonication. The supernatant was pre-cleared with a Salmon Sperm DNA/Protein A Agarose-50% Slurry. The recovered supernatant was incubated with antibodies Pu.1 (1:10, polyclonal rabbit IgG, immunogen affinity purified) or an isotype control IgG (15 µg rabbit polyclonal or 5 µg mouse monoclonal IgG, protein A purified) (Millipore) overnight at 4 °C with rotation. The antibody/DNA complex was collected using Salmon Sperm DNA/Protein A Agarose Slurry for 1 h at 4 °C with rotation, and the complex was eluted by elution buffer. Crosslinks were reversed with 5 M NaCl heating at 65 °C for 4 h. The DNA sample was then purified and measured by qRT-PCR.

### Flow cytometric analysis of apoptosis

Cell apoptosis rate was measured by using an Annexin-V–Fluoresceinisothiocyanate Apoptosis Detection Kit (Oncogene ResearchProducts, Boston, MA) that quantitatively measures the percentage of early apoptotic cells via flow cytometric analysis.

### Western blotting assay

Cell proteins were prepared using cell lysis buffer. Equal amounts of protein (50 mg) were separated by 10% sodium dodecyl sulfate-polyacrylamide gel electrophoresis and then transferred to nitrocellulose membranes (Merck Millipore) by electro-blotting. The membranes were blocked with 5% nonfat dry milk in TBST for 1 h, and then incubated with primary antibody anti-PU.1 (Abcam, Cat#:ab76543), anti-LC3B (Abcam, Cat#:ab48394), anti-p62 (Abcam, Cat#:ab56416), anti-caspase3 (Abcam, Cat#:ab13586), anti-c-caspase3 (Abcam, Cat#:ab2302), anti-PARP (Abcam, Cat#:ab32138), anti-c-PARP (Abcam, Cat#:ab32064), anti-ATG5 (Abcam, Cat#:ab227132), anti-ATG16L1 (Abcam, Cat#:ab188642) and anti-GAPDH (Abcam, Cat#:ab8245) overnight at 4 °C before subsequent incubation with second antibody (Cell Signaling Technology) for 1 h at 37 °C. Protein binds were visualized using enhanced chemiluminescence reagent (Pierce).

### TUNEL assay

Apoptosis in transplanted-tumour tissues was detected using the TUNEL assay, performed according to the guidelines recommended by the TUNEL Assay Kit (KeyGen, Nanjing, China).

### GFP-LC3 analysis

Cells were transfected with a GFP-LC3-expressing plasmid. After 24 h, cells were fixed in 3.7% formaldehyde for 20 min, washed with phosphate-buffered saline (PBS), mounted and inspected using a fluorescence microscope. A minimum of 150 GFP-positive cells were counted under each condition, and the graphs were plotted as percentage of GFP-LC3-positive cells over total transfected cell population.

### Transmission electron microscopy

Cells were fixed with a solution containing 3% glutaraldehyde plus 2% paraformaldehyde in 0.1 mol/l phosphatebuffer (pH 7.4), followed by 1% OsO_4_. After dehydration, thin sections were stained with uranyl acetate and leadcitrate for observation under a JEM 1011CX electron microscope (JEOL, USA, Inc.). Digital images were obtained using an Advanced Microscopy Techniques imaging system.

### Mice xenograft models and immunohistochemistry analysis

All animal experiments strictly followed the guidelines of the Institutional Review Board of Jinling Hospital. Approximately 5.0 × 10^6^ HepG2/miR-142-3p or HepG2/control cells were suspended in 100 μl PBS and injected subcutaneously into the right side of the posterior flank of female BALB/c athymic nude mice (Department of Comparative Medicine, Jinling Hospital, Nan Jing, China) at 5–6 weeks of age. Tumour volumes were examined every other day and were calculated using the equation: *V* = *A* × *B*^2^/2 (mm^3^), where *A* is the largest diameter and *B* is the perpendicular diameter. When the average tumour size reached approximately 50 mm^3^, sorafenib was administered via intraperitoneal injection at a dose of 30 mg/kg at one dose every other day with for three total doses. After 2 weeks, all mice were killed, and necropsies were performed. The primary tumours were excised and analysed by haematoxylin and eosin staining, immunostaining of Ki-67, ATG5 and ATG16L1, TUNEL staining and western blotting analysis for LC3 and ATG5 and ATG16L1 protein expression.

### Statistical analysis

All statistical analyses were conducted using the SPSS 17.0 statistical software and the experimental data were presented as mean ± S.D. Two-group comparisons were performed with Student's *t*-test. Multiple group comparisons were analysed with one-way analysis of variance. All tests performed were two-sided. Statistically significant negative correlation between ATG5 and miR-142-3p expression levels in HCC tissues from 38 cases was analysed by Spearman’s correlation analysis. Statistically significant positive correlation between PU.1 and miR-142-3p expression levels in HCC tissues from 38 cases was analysed by Spearman’s correlation analysis. Statistically significant positive correlation between ATG16L1 and miR-142-3p expression levels in HCC tissues from 38 cases was analysed by Spearman’s correlation analysis. *p*-Value <0.05 was considered statistically significant.

## Electronic supplementary material

Supplementary Figure 1

Supplementary Figure 2

Supplementary Figure 3

Supplementary Figure 4

Supplementary Figure legends
