# Supplementary figures and images for: PU.1/microRNA-142-3p targets ATG5/ATG16L1 to inactivate autophagy and sensitize hepatocellular carcinoma cells to sorafenib
Source: Cell Death Dis. 2018 Feb 22;9(3):312. doi: 10.1038/s41419-018-0344-0 (PMC5833744; doi:10.1038/s41419-018-0344-0)

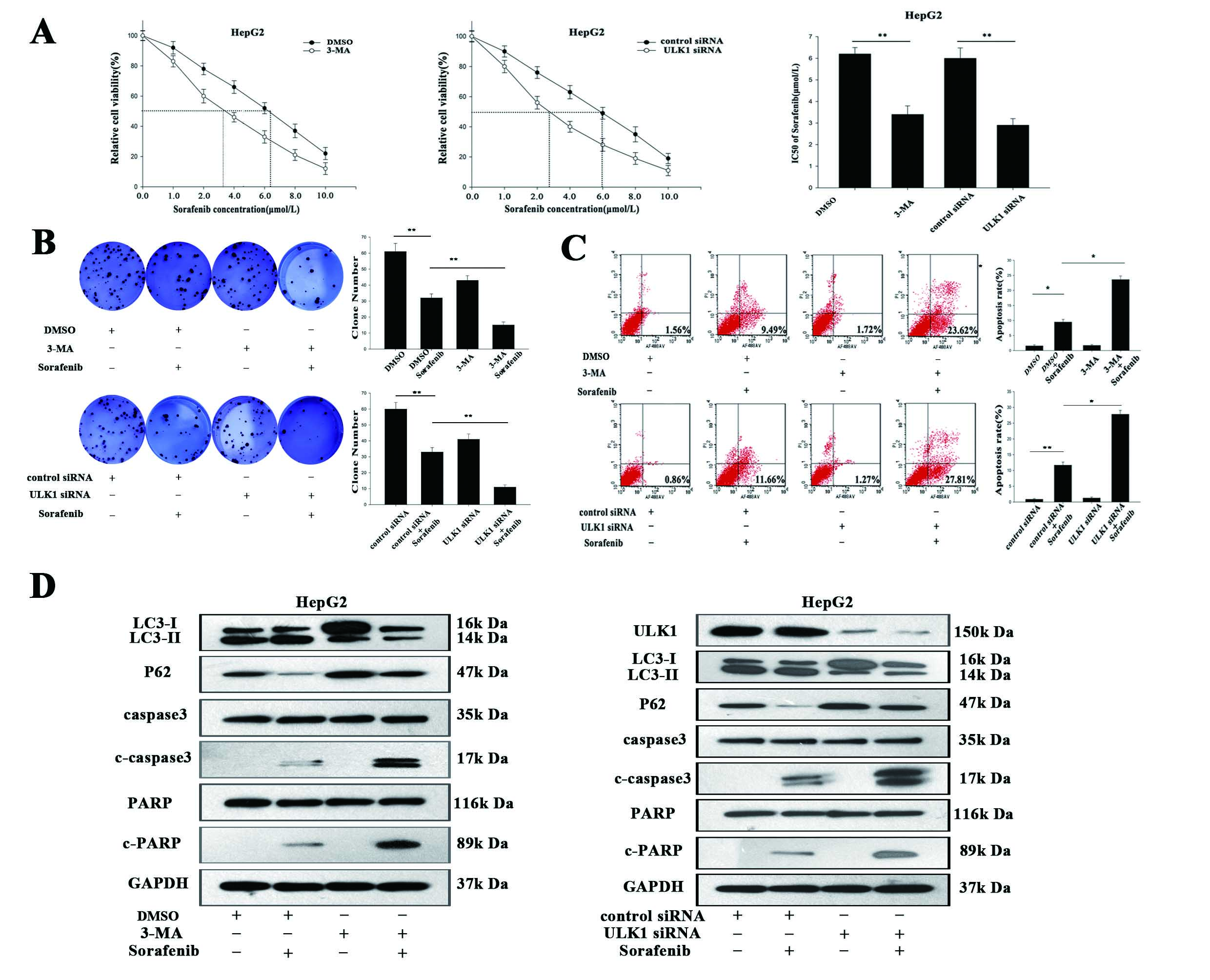

Supplement: Supplementary file 1 — Supplementary Figure 1 [file 41419_2018_344_MOESM1_ESM.jpg]

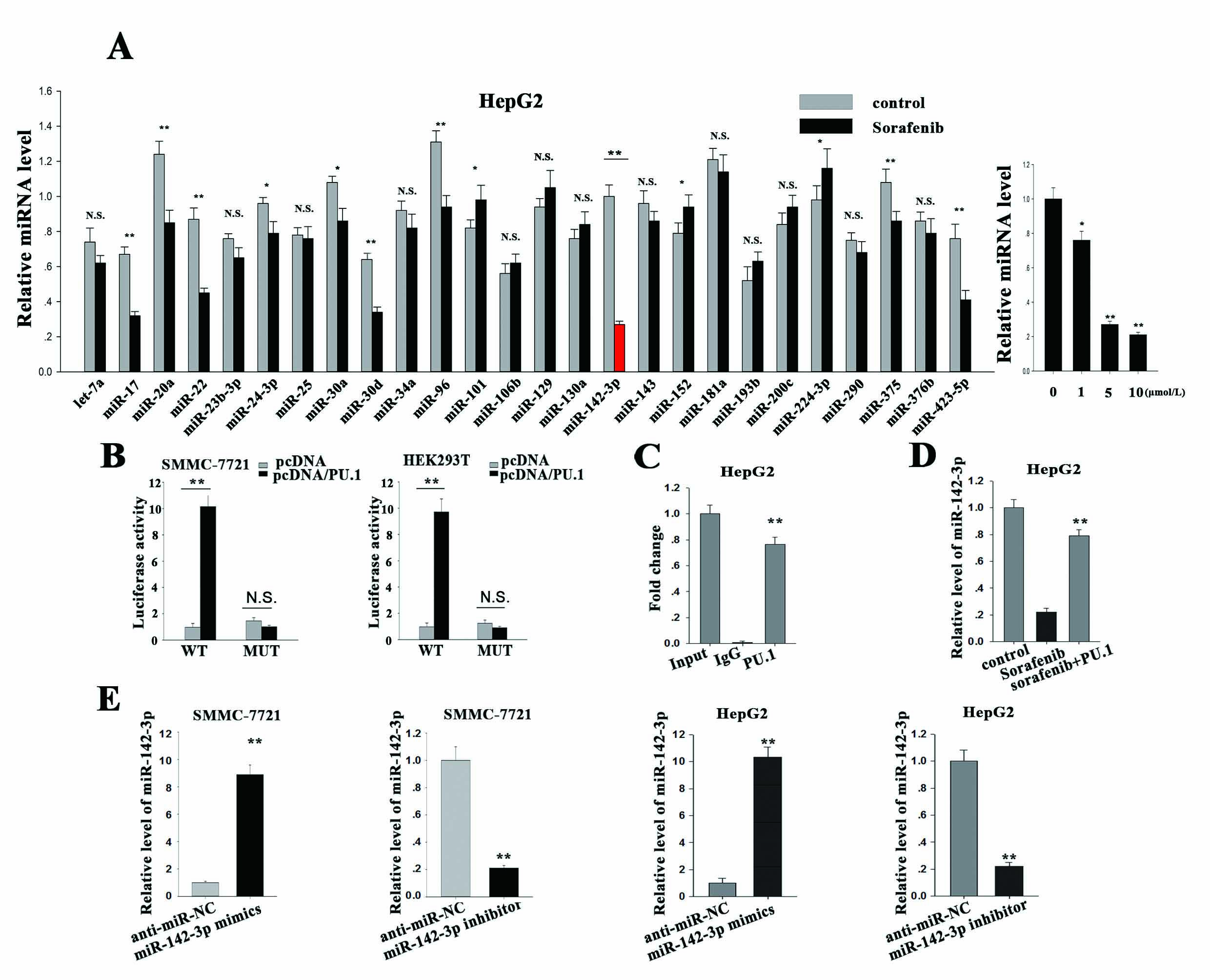

Supplement: Supplementary file 2 — Supplementary Figure 2 [file 41419_2018_344_MOESM2_ESM.jpg]

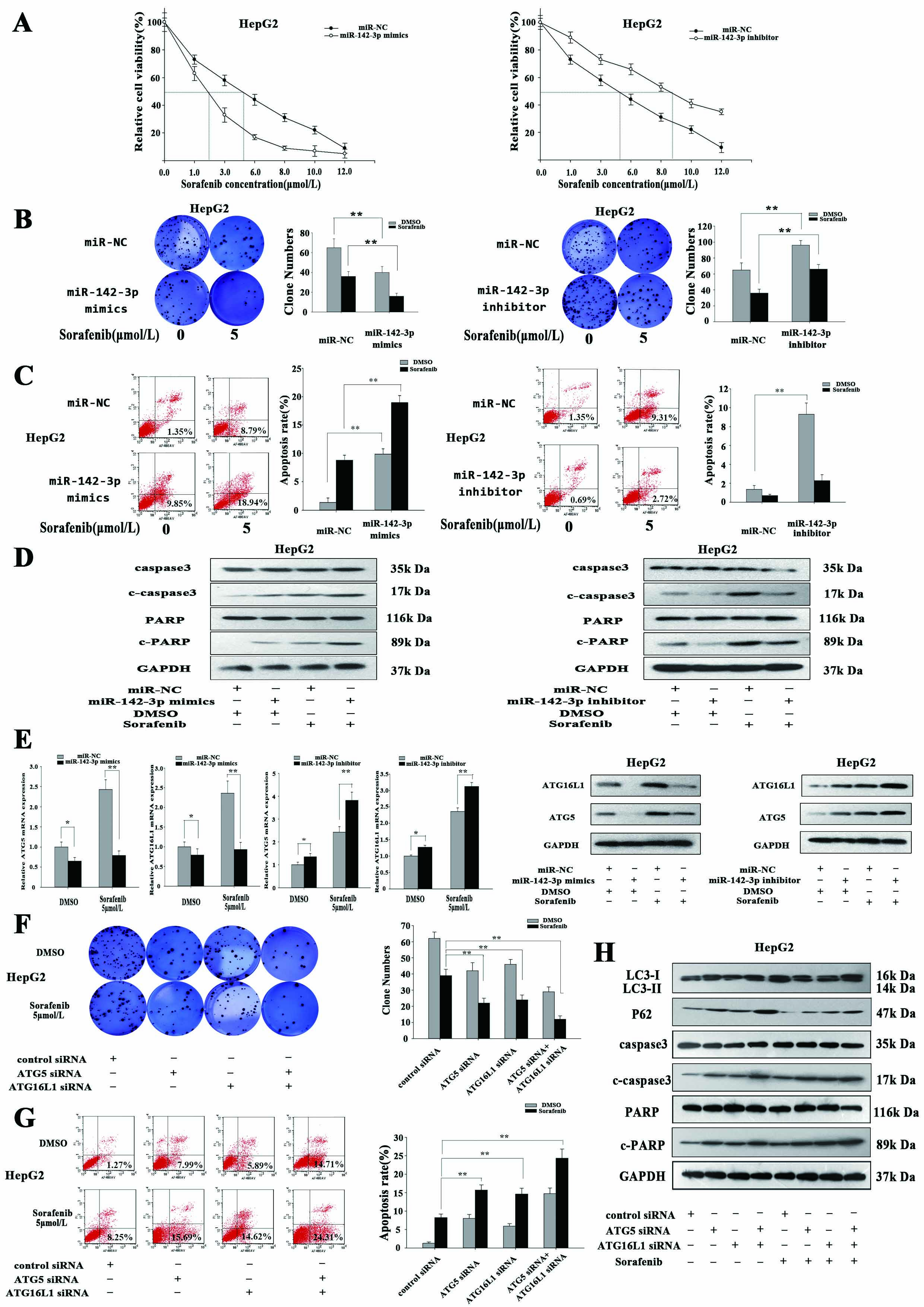

Supplement: Supplementary file 3 — Supplementary Figure 3 [file 41419_2018_344_MOESM3_ESM.jpg]

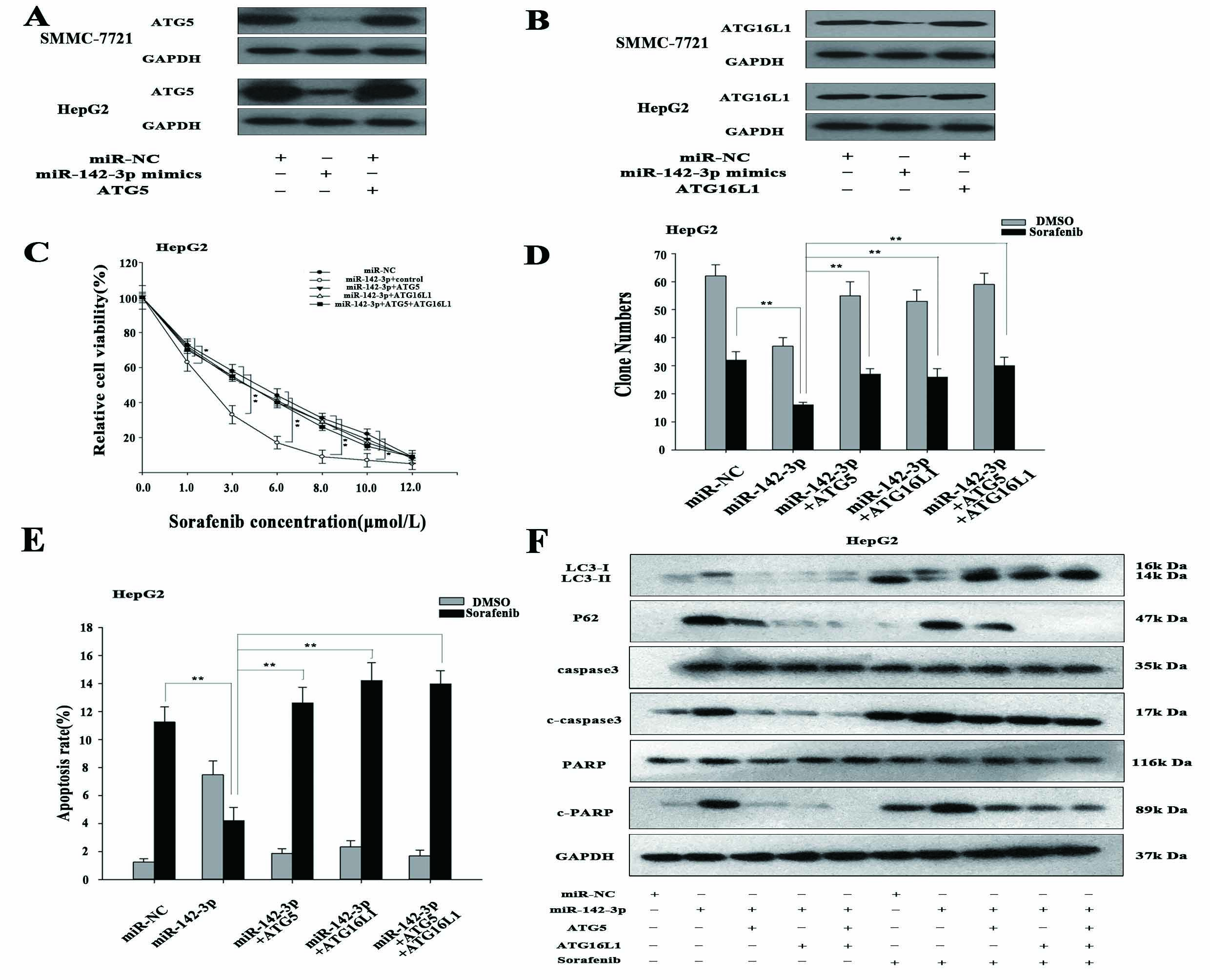

Supplement: Supplementary file 4 — Supplementary Figure 4 [file 41419_2018_344_MOESM4_ESM.jpg]
