# Supplementary material for: PU.1/microRNA-142-3p targets ATG5/ATG16L1 to inactivate autophagy and sensitize hepatocellular carcinoma cells to sorafenib
Source: Cell Death Dis. 2018 Feb 22;9(3):312. doi: 10.1038/s41419-018-0344-0 (PMC5833744; doi:10.1038/s41419-018-0344-0)
Supplement: Supplementary file 5 — Supplementary Figure legends [file 41419_2018_344_MOESM5_ESM.doc]

**Supplementary Figure 1. Suppression of autophagy-mediated enhancements of HCC cell sensitivity to sorafenib.**

**A.** MTT assays were employed to measure the relatively viability of HepG2 cells treated with sorafenib after treatment with 3-MA or ATG1/ULK1 silencing. **B-E.** Flow cytometry and western blotting were performed to measure the apoptosis rate and apoptosis (c-caspase3 and c-PARP)- and autophagy-related protein levels. All data are presented as the mean ± S.D. from three independent experiments. The p-values represent comparisons between groups (*p < 0.05, **p < 0.01).

**Supplementary Figure 2. MiR-142-3p may be upregulated by PU.1 in HCC cells.**

**A.**qRT-PCR was used to assess autophagy- and apoptosis-related miRNA levels in HepG2 cells treated with sorafenib **B.** ChIP assays performed in HepG2 cells. **C.** Rescue assays were performed to confirm the effect of PU.1 on miR-142-3p expression. **D.** Luciferase reporter assays was applied to determine the effect of PU.1 on the promoter of miR-142-3p in SMMC-7721 cells and HEK293T cells. **E.** The transfection efficiency of miR-142-3p inhibitor and mimics was evaluated by qRT-PCR in SMMC-7721 cells and HepG2 cells.

**Supplementary Figure 3. Forced expression of miR-142-3p re-sensitized HepG2 cells to sorafenib by inhibiting autophagy, and ATG5 and ATG16L1 are direct targets of miR-142-3p.**

**A-B.** MTT and colony formation assays were performed to measure the influence of miR-142-3p on the cytotoxicity of different concentrations of sorafenib and HepG2 cell proliferation. C. Flow cytometric analysis was employed to determine the effect of miR-142-3p on apoptosis rates and apoptosis-related protein levels in HepG2 cells treated with different concentrations of sorafenib. D. Western blotting was applied to assess apoptosis- and autophagy-related protein levels. All data are presented as the mean ± S.D. from three independent experiments. The p-values represent comparisons between groups (*p < 0.05, **p < 0.01). **E.** qRT-PCR and western blotting were used to measure ATG5 and ATG16L1 mRNA and protein levels in HEPG2 cells transfected with miR-142-3p mimics or inhibitors and treated with or without sorafenib. **F-G.** Colony formation and flow cytometric assay were employed to measure the effects of ATG5 and ATG16L on proliferation and apoptosis in HEPG2 cells treated with or without sorafenib. **H.** Western blot assay was performed to measure the apoptosis- and autophagy-related protein levels. All data are presented as the mean ± S.D. from three independent experiments. The p-values represent comparisons between groups (*p < 0.05, **p < 0.01).

**Supplementary Figure 4. The effects of miR-142-3p on sorafenib resistance are dependent on the regulation of ATG5 and ATG16L1.**

**A-B.** Western blotting was utilized to assess the levels of the proteins ATG5 and ATG16L1 in SMMC-7721 and HepG2 cells . **C.** MTT assay was performed to measure the sensitivity of HepG2 cells co-transfected with miR-142-3p mimics and ATG5 or ATG16L1 to sorafenib. **D.** Colony formation assay was employed to determine the proliferation ability of HepG2 cells co-transfected with miR-142-3p mimics and ATG5 or ATG16L1. **E.** Flow cytometry assay was employed to measure the apoptosis rate in HepG2 cells co-transfected with miR-142-3p mimics and ATG5 or ATG16L1. **F.** Western blotting was utilized to assess the levels of the apoptosis-related proteins c-caspase-3 and c-PARP and autophagy-related proteins in HepG2 cells co-transfected miR-142-3p mimics and ATG5 or ATG16L1. All data are presented as the mean ± S.D. from three independent experiments. The p-values represent comparisons between groups (*p < 0.05, **p < 0.01).
